# Neuromuscular diseases with hypertrophic cardiomyopathy

**DOI:** 10.21542/gcsp.2018.27

**Published:** 2018-08-12

**Authors:** Sergi Cesar

**Affiliations:** Arrhythmia, Inherited Cardiac Diseases and Sudden Death Unit, Pediatric Cardiology Department, Sant Joan de Déu Hospital and Sant Joan de Déu Research Institute, University of Barcelona, Barcelona, Spain

## Introduction

Neuromuscular disorders are frequently associated with cardiac abnormalities, even in pediatric population^1^. Cardiac involvement includes both structural changes and conduction disease. In general, HCM is a rare manifestation of neuromuscular diseases^2^.

Autosomal dominant inheritance with mutations in sarcomeric genes are described in about 60% of young adults and adult population with HCM. Other genetic disorders, such as inherited metabolic and neuromuscular diseases and other chromosome abnormalities are responsible of 5–10% of HCM in adults^3^. We review the most frequent neuromuscular diseases related with HCM.

## Mitochondrial dysfunction

Often, mitochondrial diseases in newborns and infants can lead to heart, skeletal muscle and central nervous system abnormalities due to, in most cases, alterations in nuclear DNA^4^. Late onset manifestations are related with single-organ affectation in adults and mtDNA mutations are most frequent than nuclear DNA mutations^5,6^. Concerning cardiac phenotype, concentric HCM with rapid evolution to dilated and hypokinetic cardiomyopathy is frequent within mitochondrial diseases^4^. Genetics and skeletal biopsy is mandatory in patients with mitochondrial disease suspicion^7^. Early encephalopathy in infants and cardiomyopathy are associated with a worst prognosis^4,6,7^. There is no specific treatment for this group of diseases, but some agents have being studied for treating mitochondrial diseases (agents increasing electron transfer chain function, energy buffer, antioxidants, restoration agents of nitric oxide production, cardiolipin protectors and agents enhancing mitochondrial biogenesis)^8^. In patients with CoQ10 deficiency, ubiquinone can improve both electronic transfer chain function and clinical manifestations^7^.

### Friedreich’s ataxia

Friedreich’s ataxia (FA) is a multisystem autosomal recessive disease involving mitochondrial function due mutations in *FXN* gene, located on chromosome 9q, which encodes a 210 amino acid Frataxin protein. GAA triplet repeat expansion in intron 1 of FXN occurs in 96–98% of FA patients, with alleles containing 66 to 1300 GAA triplet repeats^9^. Earlier disease is related with larger numbers of GAA repeats and more rapid disease progression^10^.

These abnormalities generate oxidative cellular stress and enzyme deficiency due to iron-sulfur clusters, which is the cause of respiratory chain dysfunction^11^. FA is characterized by progressive limb and gait ataxia, but other features such as spasticity absent lower limb reflexes, impaired vibration sense and proprioception, scoliosis have been described. HCM is a very frequent finding and heart failure is the most common cause of death. Patient with FA and HCM have an early onset within the first or second decades with a poor correlation with the neurological level of disability^12,13^.

Histologically, left ventricle cellular hypertrophy, diffuse fibrosis and focal myocardial necrosis have been described^12^.

**Figure 1.**
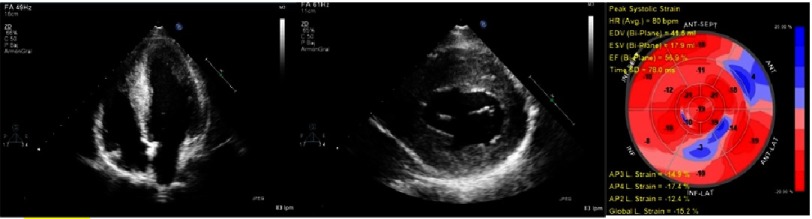
Echocardiography from a FA patient with HCM. The images show an apical 4 chamber with left ventricle HCM (mid-septal and apical regions) and left ventricle short axis image with concentric HCM. The last image shows regional dysfunction and decreased longitudinal strain analyzed with speckle-tracking myocardial strain.

Echocardiographic hallmark is a concentric LV hypertrophy with absence of left ventricular outflow tract obstruction, but eccentric hypertrophy might be present (Figure 1)^13–15^.

**Figure 2.**
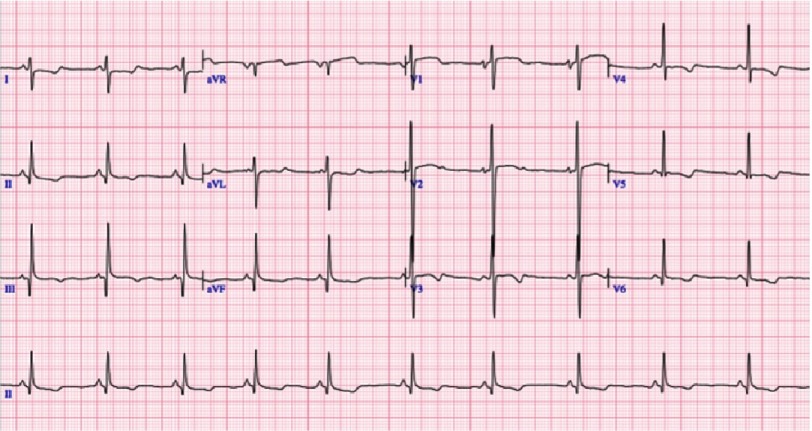
FA patient with HCM. ECG shows LVH data and repolarization abnormalities.

Diastolic function is mildly impaired, with pseudo-normal diastolic pattern described in some series. Contrary to other diseases that cause concentric hypertrophy pattern with a sparkling granular texture (e.g., amyloidosis), atrial enlargement and pericardial effusion are rare in FA^3,13^. LV fibrosis is described and has been related with progressive LV thinning and dilatation^16^. Despite left ventricular ejection fraction being preserved in many patients, regional myocardial analysis with speckle-tracking can show regional dysfunction or decreased values of global longitudinal strain, as reported previously^17,18^. End-stage patients with FA can develop a reduced ejection fraction with hypokinesia and slight LV dilatation (Figure 2)^11^.

The QRS duration in most FA patients, is normal even with significant LV hypertrophy. T-wave abnormalities are very frequent, especially in left precordial leads^13^. Supraventricular arrhythmias such as AV reentry tachycardia, atrial fibrillation and atrial flutter are described^12,19^.

There is no specific treatment for HCM in FA patients. Management of heart failure symptoms (salt restriction, diuretic therapy), ACE inhibitors or angiotensin II receptor blockers may be beneficial in long-term treatment^20^. Treatment of atrial arrhythmias is mandatory, because the important atrial role to LV filling and cardiac output^14^. The drug idebenone acts as a transporter in the electron transport chain and has been advocated for use in FA following studies showing mild diastolic improvement and reduction LVH^21,22^. However, further trials have shown no benefit.

Cardiac transplantation is not commonly performed, due to advanced impairment of both motor skills and muscle strength.

### Barth syndrome and other 3-methylglutaconic (3-MGA) aciduria disorders

Barth syndrome (type II 3-MGA-aciduria), is characterized with skeletal myopathy, neutropenia, growth retardation and 3-metylglutaconic aciduria. It is associated with both HCM/LVNC and DCM phenotypes.^8,23^.

**Figure 3.**
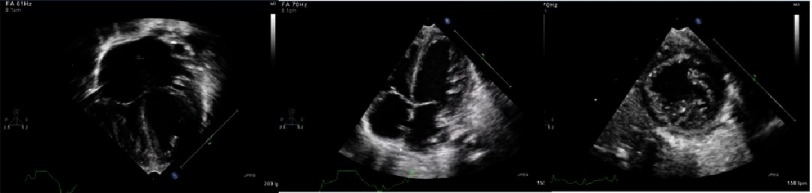
Echocardiography of a newborn diagnosed with Barth syndrome. 1) 4 chamber view shows biventricular hypertrophy, 2) shows mixed non-compaction and LV hypertropphy and 3) short axis view shows postero-apical non compaction.

Barth syndrome is an X-linked autosomal recessive disease caused by *TAZ* gene mutations. This gene encodes for tafazzin, an acyl-transferase that catalyzes cardiolipin remodelling in the inner mitochondrial membrane^24^. Barth syndrome causes heart failure, arrhythmias and sepsis in male newborns and infants (Figure 3)^23^.

Other 3-methylglutaconic aciduria diseases related with HCM are TMEM70 mutations (type IV 3-MGA-aciduria)^25^.

Sengers syndrome presents in two forms of the disease - a lethal neonatal form characterized by severe HCM, cataract, skeletal myopathy, lactic acidosis^25–27^ and a more benign adult form. It is caused by mutations in AGK gene that encodes for the mitochondrial acylglycerol kinase.

### Respiratory chain-related disorders

Respiratory chain disorders are clinically and genetically heterogenous group. Gene mutations of complex-I respiratory chain subunits in mtDNA and nuclear DNA have been related with HCM.^4,5^ Patients could have epilepsy, ataxia, muscle weakness, neurosensorial deafness, lactic acidemia and hypoglycemia^28^.

Other mutations in II-complex respiratory chain subunits in nuclear DNA have been related with HCM, DCM and LVNC. Mutations have also been identified in SDH genes (*SDHA* and *SDHD* genes). Muscular weakness, ataxia, seizures, ophthalmoplegia, pigmentary retinitis, optic atrophy and lactic acidosis are manifestations of these patients^29^.

Stroke-like episodes, epilepsy, hypoglycemia, lactic acidosis and optic atrophy have been related with or without HCM, DCM and histiocytoid cardiomyopathies in cases of mutations in *MTCYB* gene that encodes cytochrome-b protein in III-complex respiratory chain^30^.

Finally, other disorders related with IV-complex respiratory chain have been associated with HCM, DCM and histiocytoid cardiomyopathy. Genes encoding subunits of IV-complex in mtDNA and nuclear mtDNA, such as *COX6B*, and genes encoding assembly factors in the IV-complex respiratory chain, such as *COX10*, *SCO1*, *COA6* and *SURF*, have been described^31,32^.

### tRNA and rRNA-related disorders

Mitochondrial tRNA gene mutations cause HCM, DCM and histocytoid cardiomyopathy with or without multiorgan involvement. MERRF (Myoclonus epilepsy and ragged red fibers) and MELAS (mitochondrial myopathy, encephalopathy, lactic acidosis and stroke-like episodes), due to mutations in *MTTK* and *MTTL1* genes respectively, are examples of this group^33,34^. Mutations in *MTRNR2* gene, encoding mitochondrial ribosome protein 16S, have been related with HCM^35^. Other mutations in rARN genes, such as *MRPL44*, and mutations in *TSFM*, have been related with HCM and multiorgan syndrome^36,37^.

### Mitochondrial depletion DNA syndromes

These disorders have in common a significant drop in mitochondrial DNA in affected tissues. Mutations in *TYMP* (also called *ECGF1*) gene can lead to reduced levels of thymidine phosphorylase enzyme activity, which is found in Mitochondrial Neurogastrointestinal Encephalopathy (MNGIE). Clinical manifestations of MNGIE are progressive gastrointestinal dysmotility, cachexia, ptosis/ophthalmoplegia, leukoencephalopathy, demyelinating peripheral neuropathy. There is no hard evidence for HCM but ECG analysis showed left ventricular hypertrophy in some cases^38^.

### CoQ10 biosynthesis deficiency

Mutations in genes encoding biosynthesis of CoQ10 (*COQ2*, *COQ9* and *PDSS1* genes) can lead to CoQ10 deficiency, that can be related with encephalopathy, skeletal myopathy, ataxia and nephrotic syndrome. Isolated HCM or associated with other multi-organ affectation have been associated with mutations in *COQ2*, *COQ4*, *COQ9* genes^39^.

## X-linked recessive muscular dystrophies

### Dystrophinopathies

The most frequent X-linked muscular dystrophies are Duchenne muscular dystrophy (DMD) and Becker muscular dystrophy (BMD)^40^. Mutation in the *DMD* gene leads to an absence of functional protein in DMD, whereas BMD shortened dystrophin or reduced amount is detected. Weakness of leg, pelvic and shoulder girdle muscles starts in early childhood. Cardiac involvement in BMD may precede the skeletal muscle weakness. Dilated cardiomyopathy is the final cardiac phenotype, but hypertrophic phenotype is described within female carriers of dystrophinopathy and diastolic dysfunction followed by eccentric hypertrophy are described^41–43^. There are few reports in BMD patients with hypertrophic cardiomyopathy^44^. Abnormal circumferential strain is described in DMD patients despite normal ejection fraction and pre-symptomatic stage^45^. Cardiovascular complications are a leading cause of morbidity and mortality in DMD patients^41^.

**Figure 4.**
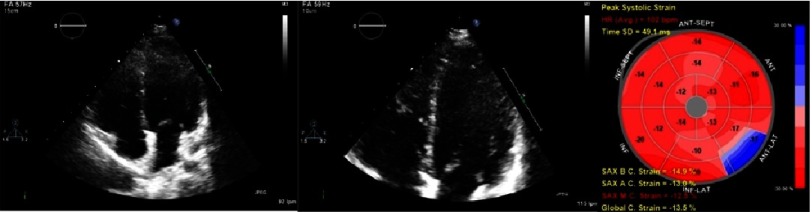
Echocardiography from a pediatric Duchenne muscular dystrophy patient. Note the left ventricle image with slight thickened interventricular septum and heterogeneous echogenicity. The last image shows regional dysfunction and decreased global circumferential strain based on speckle-tracking.

**Figure 5.**
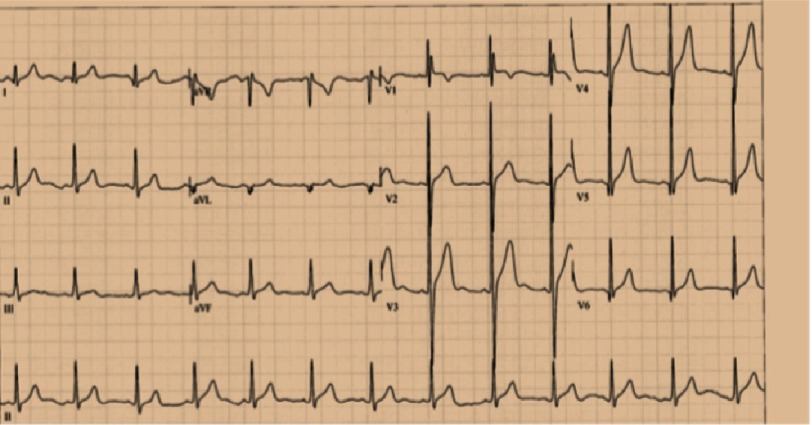
BMD patient with cardiomyopathy. ECG shows LVH data, short PR interval and J point elevation in V2-V4 leads.

There is no specific treatment for cardiomyopathy in dystrophinopathies (Figures 4 and 5). Some evidence suggests cardiac benefits with early treatment with ACE inhibitors, improving long-term cardiac outcomes^41^.

### Emery-Dreifuss muscular dystrophy

Emery-Dreifuss muscular dystrophy (EDMD) is a rare hereditary disease characterized by early joint contractures of Achilles tendons, elbows and rigid spine, childhood onset of muscle weakness and wasting and adult-onset cardiac disease (arrhythmias, cardiomyopathy). EDMD can be transmitted X-linked (mutations in *EMD* gene) or autosomal (mutations in *LMNA* gene). Mutations in *EMD* gene can lead to abnormalities in emerin protein, which is a component of the nuclear envelope^46,47^. Other mutations in *FHL1* gene are described in some families with X-linked EDMD, some of them with HCM^46^.

## Myotonic dystrophies

### Myotonic dystrophy type 1

Myotonic dystrophy type 1 (DM-1), or Steinert disease, is a genetic disease due to an expansion of CTG triplet in *DMPK* gene on chromosome 19. DM-1 is multisystemic disease with autosomal dominant transmission and incomplete penetrance. Myotony and muscle weakness are the main clinical manifestations but both cardiovascular and respiratory system are also involved^48^. Arrhythmias are the second cause of death in DM-1 patients, most of them suffering sudden cardiac death^49^. About cardiac involvement, concentric HCM are described in these patients, mainly detected in adults. Other structural findings are DCM and LVNC^50^.

## Myofibrillar myopathies

Myofibrillar myopathies (aggregate myopathies) are a genetically heterogeneous diseases with manifestation in both skeletal and cardiac muscle. Focal dissolution of myofibrils and aggregation of degraded myofibrillar products into inclusions containing desmin and other proteins have been found close to Z-disc.

Mutations in desmin (*DES*), alpha-aB crystallin (*CRYAB*), myotilin (*MYOT*), Z band alternatively spliced PDZ-containing protein (*ZASP*), filamin C (*FLNC*) and Bcl-2-associatged athanogene-3 (*BAG3*) are responsible for different phenotypes of myofibrillar myopathy^53^.

The most common myofibrillar myopathy is caused by *DES* gene mutations (Desminopathy) and typically cause skeletal myopathy and cardiomyopathy (dilated and restrictive cardiomyopathy, HCM, AV block and arrhythmogenic cardiomyopathy are also described)^51^.

AlphaB-crystallinopathy is an infrequent subtype of myofibrillar myopathy, caused by a mutation in *CRYAB* gene, and clinically characterized by proximal upper limb and distal lower limb weakness, velopharyngeal muscles, respiratory failure, HCM and lens opacities^52^.

Myotilinopathy is caused by mutations in *MYOT* gene and is characterized by late-onset disorder with distal weakness of lower limbs or limb-girdle weakness. Peripheral neuropathy, respiratory failure and HCM are rare associated findings^52^.

ZASPopathy can be related with very late-onset symptoms, similar to myotilinopathies, but peripheral neuropathy and HCM have been described^52^. Filaminopathy related with myofibrillar myopathy is an adult-onset proximal weakness with both respiratory and cardiac abnormalities^52^. BAG3 mutations associated with myofibrillar myopathy is a rare disease characterized by rapid progressive limb and axial muscle weakness, HCM and respiratory insufficiency^52^.

## Other rare neuromuscular disorders related with HCM

### Limb girdle muscular dystrophy

Limb girdle muscular dystrophies are a group of neuromuscular disorders characterized by proximal muscular weakness and wasting of the arms and legs. Within autosomal recessive inheritance group, HCM with or without skeletal muscle manifestations has been described in Limb Girdle 1C (autosomal dominant inheritance, mutation in *CAV3* gene) and 2J (autosomal recessive inheritance, mutation in *TTN* gene)^53,54^.

### Facioscapulohumeral muscular dystrophy

Facioscapulohumeral muscular dystrophy (FSHMD) affects facial, shoulder girdle, and sometimes peroneal muscles. FSHMD is caused by a deletion of an integral number of 3.3kb tandem repeats from the subtelomeric region on chromosome 4q35. FSHMD has been related with HCM in some publications^51,55^.

### Congenital myopathies

Within congenital myopathies, HCM has been described in Nemaline myopathy type 3, related with mutations in Alpha-actin, alpha tropomyosin and nebulin gene with a general autosomal recessive inheritance pattern^56^. Multiminicore disease (rigid spine syndrome), an autosomal recessive disease related with ryanodine receptor gene and selenoprotein N1 gene mutations, has been associated with HCM and RCM^53,56^.

### Primary carnitine deficiency

Classic initial presentation of primary carnitine deficiency is hypoketotic hypoglycemic encephalopathy, with hepatomegaly, elevation of transaminases and hyperammonemia. Muscle weakness and cardiomyopathy can be present in these patients, which could be both dilated or hypertrophic cardiomyopathy. Mutations in *SLC22A5* gene have been related with primary carnitine deficiency^57^.
